# Supplementary figures and images for: Pregnancy outcomes after hysteroscopic surgery in women with cesarean scar syndrome
Source: PLoS One. 2020 Dec 3;15(12):e0243421. doi: 10.1371/journal.pone.0243421 (PMC7714235; doi:10.1371/journal.pone.0243421)

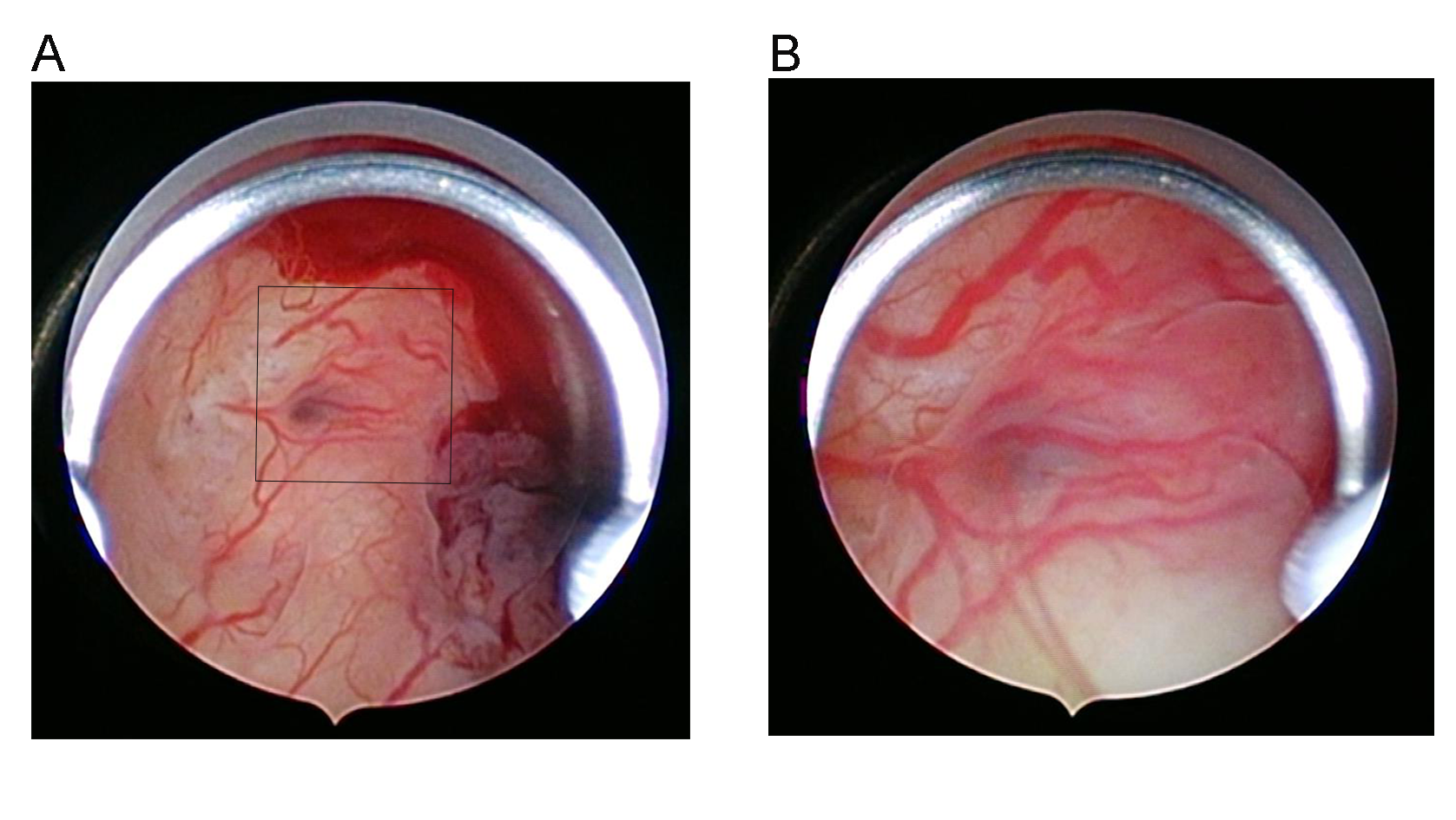

Supplement: S1 Fig — (B) Enlargement of the area indicated with the square in (A). (TIF) [file pone.0243421.s001.tif]
